# Differential diagnostic value of rheumatic symptoms in patients with Whipple’s disease

**DOI:** 10.1038/s41598-021-85217-2

**Published:** 2021-03-16

**Authors:** Gerhard E. Feurle, Verena Moos, Andrea Stroux, Nadine Gehrmann-Sommer, Denis Poddubnyy, Christoph Fiehn, Thomas Schneider

**Affiliations:** 1 DRK Krankenhaus Neuwied, Eduard Moerikestrasse 12, 56567 Neuwied, Germany; 2 Medizinische Klinik I für Gastroenterologie, Infektiologie und Rheumatologie, Charité-Universitätsmedizin Berlin, Berlin, Germany; 3 Institut für Biometrie und Klinische Epidemiologie, Charité-Universitätsmedizin Berlin, Berlin Institute of Health, Berlin, Germany; 4 Praxis für Rheumatologie, Klinische Immunologie, Medical Center, Baden-Baden, Germany

**Keywords:** Diseases, Rheumatology, Signs and symptoms

## Abstract

Most patients with Whipple’s disease have rheumatic symptoms. The aim of our prospective, questionnaire-based, non-interventional clinical study was to assess whether these symptoms are useful in guiding the differential diagnosis to the rheumatic disorders. Forty patients with Whipple’s disease, followed by 20 patients for validation and 30 patients with rheumatoid-, 21 with psoriatic-, 15 with palindromic- and 25 with axial spondyloarthritis were recruited for the present investigation. Patients with Whipple’s disease and patients with rheumatic disorders were asked to record rheumatic symptoms on pseudonymized questionnaires. The data obtained were subjected to multiple logistic regression analysis. Episodic pain with rapid onset, springing from joint to joint was most common in patients with palindromic arthritis and second most common and somewhat less conspicuous in Whipple’s disease. Continuous pain in the same joints predominated in patients with rheumatoid-, psoriatic-, and axial spondyloarthritis. Multiple logistic equations resulted in a predicted probability for the diagnosis of Whipple’s disease of 43.4 ± 0.19% (M ± SD) versus a significantly lower probability of 23.8 ± 0.19% (M ± SD) in the aggregate of patients with rheumatic disorders. Mean area under the curve (AUC) ± SD was 0.781 ± 0.044, 95% CI 0.695–0.867, asymptotic significance p < 0.001. The logistic equations predicted probability for the diagnosis of Whipple’s disease in the initial series of 40 patients of 43.4 ± 0.19% was not significantly different in the subsequent 20 patients of 38.2 ± 0.28% (M ± SD) (p = 0.376). The data may be useful in a predictive algorithm for diagnosing Whipple’s disease. The project is registered as clinical study DRK S0001566.

## Introduction

From 2013 to 2015, an estimated 54.4 million US adults (22.7%) annually were informed by a doctor that they had some form of rheumatic disease, such as rheumatoid arthritis, gout, lupus, fibromyalgia etc^1^. One of the disorders with rheumatic signs and symptoms is Whipple’s disease, a rare disease with an estimated prevalence of one in a million^2^. Identifying a patient with Whipple’s disease arthritis among the many others with rheumatic symptoms is like the proverbial search for a needle in a haystack. As the predictive value of a diagnostic test, i.e. the percentage of positive tests that are true positives depends on the prevalence of the disease, the rate of false positive test results will be high when prevalence is so low. It is not surprising therefore that it may take years to diagnose Whipple’s disease among the many other disorders with rheumatic symptoms^3–6^.

In 1907, G.H. Whipple described the first patient with this disease now bearing his name: “The first symptoms were attacks of arthritis coming on in various joints. They were transient, the first lasting but six to eight hours. These recurred again and again three to four times a week in damp weather, once a week perhaps in dry weather, lasting from six to twenty four hours; nearly every joint has been affected. Sometimes the joints were hot, swollen, and tender; at other times only painful. … pain might seem to be in the muscles …”^2^.

Later, Kelly and Weisiger reviewed 95 cases of Whipple’s disease in the English literature through 1961 and three cases seen by the authors in 1963 and found “the arthropathy to be migratory in 17 instances and in at least 24 the process occurred in an intermittent or episodic fashion. The attacks usually were acute in onset but transient, extending from a few hours to several years, but most often just a matter of days”^3^.

In 2013, Krol and de Meijer entitled their letter to the editor “Palindromic rheumatism: consider Whipple’s disease”^7^. In other recent contributions, the “palindromic” character of Whipple’s disease arthritis was not mentioned^8,9^.

It is the aim of the present prospective study to compare the rheumatic symptoms of patients with Whipple’s disease with the symptoms of patients diagnosed with rheumatoid arthritis, psoriatic arthritis, axial spondyloarthritis, and palindromic arthritis.

It may seem obsolete to record traditional clinical symptoms in the ‘platinum age’ of rheumatology when understanding the molecular pathogenesis is leading to innovative strategies of diagnosis and treatment each year^10^. Similar to the rheumatic disorders, however, signs and symptoms are elementary for diagnosis in Whipple’s disease and even more so as noninvasive laboratory tests, such as rheumatoid factors, to base a diagnosis are not available. Guidance by signs and symptoms is critical.

## Patients, material and methods

This diagnostic study has been prepared according to the STARD 2015 reporting recommendations^11^.

Patients were prospectively recruited in the real world of two specialized hospitals in Germany, the Charité Universitätsmedizin Berlin, Medizinische Klinik I, national reference center for patients with Whipple’s disease in Berlin and the Acura Rheumazentrum, competence center for rheumatology and autoimmune diseases in Baden-Baden.

Patients from Berlin and Baden-Baden meeting the respective diagnostic criteria and being capable of reading German texts were eligible for recruitment.

In Berlin, a predetermined number of patients with Whipple’s disease was recruited, diagnosed according to established criteria^12,13^. Patients with isolated *T. whipplei* endocarditis were not registered, as rheumatic symptoms generally are lacking.

These patients in Berlin, diagnosed with Whipple’s disease and the patients in Baden-Baden, diagnosed with defined rheumatic disorders, (details see below), were requested to answer in writing a questionnaire concerning the symptoms of their disease during February 2014 to June 2016, while the target sample sizes of initially 40 patients with Whipple's disease and 30 patients with rheumatoid arthritis were reached. 48 patients with Whipple's disease had initially been registered, but eight could not be included: three did not give written informed consent and five did not return the questionnaire. The initial study group of patients with Whipple's disease, therefore, consisted of 40 patients. 25 subsequent patients with Whipple's disease were registered in Berlin for validation during the time period of July/2016 to July/2018, but five of them could not be included. Three did not consent and two did not return the questionnaire. The validation group of patients with Whipple's disease, therefore, consisted of the target number of 20 patients. The recruitment rate was lower than 100% in these patients but in no case due to selected patient characteristics.

In Baden-Baden, patients with rheumatoid arthritis, diagnosed according to the 2010 American College of Rheumatology (ACR) and European League Against Rheumatism classification criteria^14^, patients with psoriatic arthritis diagnosed according to the CASPAR criteria^15,16^, and patients with axial spondyloarthritis, diagnosed according to the ASAS classification criteria^17,18^ were recruited during the time period of February 2014 to June 2016 as in Berlin. Palindromic arthritis was diagnosed when episodic and saltatory arthritis with localized articular pain and swelling occurred in 14 patients in the presence of either anti-citrullinated protein antibodies (ACPA) or an isolated positive rheumatoid factor found in one patient.

All patients recruited for the study were asked to answer in writing a questionnaire concerning symptoms of their disease (See the “Supplementary material” for a translation in English). The patient’s diagnosis on the questionnaires remained masked after data collection, during extraction and statistical evaluation.

All questionnaires were filled out by the patients without professional assistance. The questionnaires were pseudonymized and extracted in Neuwied in Excel tables for statistical analysis.

### Statistical analysis

Statistical analysis was performed using SPSS version 25 and Graph pad prism version 8.4.3. Descriptive parameters were presented as absolute and relative frequencies for categorical variables and in terms of mean and standard deviation for quantitative measurements. Accordingly, for univariate group comparisons, Pearson’s chi-squared or the Mann–Whitney-U test was used. Column analysis with multiple comparisons were performed by one-way ANOVA followed by Holm–Sidak’s multiple comparison tests. In order to identify characteristics independently discriminating Whipple’s disease from the other rheumatic disorders, multiple logistic regression models with forward and backward selection were performed, with Whipple’s disease/other as dependent variable and characteristics significantly associated with Whipple’s disease in the univariate analyses as independent variables. We first performed univariate chi-squared tests to identify possible candidates for the multiple logistic regression analyses; only variables significantly discriminating Whipple’s disease from the respective differential diagnosis were included into the variable selection. Final logistic equation predicted values were calculated and compared using the Mann–Whitney U test. For quantification of the discriminating quality of selected characteristics, ROC analysis was performed, including AUC with 95% confidence interval. In a last step, the logistic equation obtained in the original series of patients was applied to the validation series of 20 subsequent patients with Whipple’s disease and the resulting predictors were compared using the Mann–Whitney U test. All tests were two-sided on the 0.05 level without Bonferroni correction.

### Ethics approval and consent to participate

The study protocol was approved by the Ethics Committee of the Ärztekammer Rheinland-Pfalz in Mainz, Germany; reference number: 837.498. 14 (9737) in 2014, amended for the validation group 2018-13718_1 in 2018. Each patient signed a consent form and was free to fill out the questionnaire; a few did not consent or did not return the questionnaire. Written informed consent was obtained from each subject, and all procedures were performed in accordance with the Declaration of Helsinki.

### Guidelines and regulations

The project complies with all relevant guidelines and regulations.

### Consent for publication

All Authors give their consent to publish the manuscript in “Scientific reports”.

## Results

**Figure 1:**
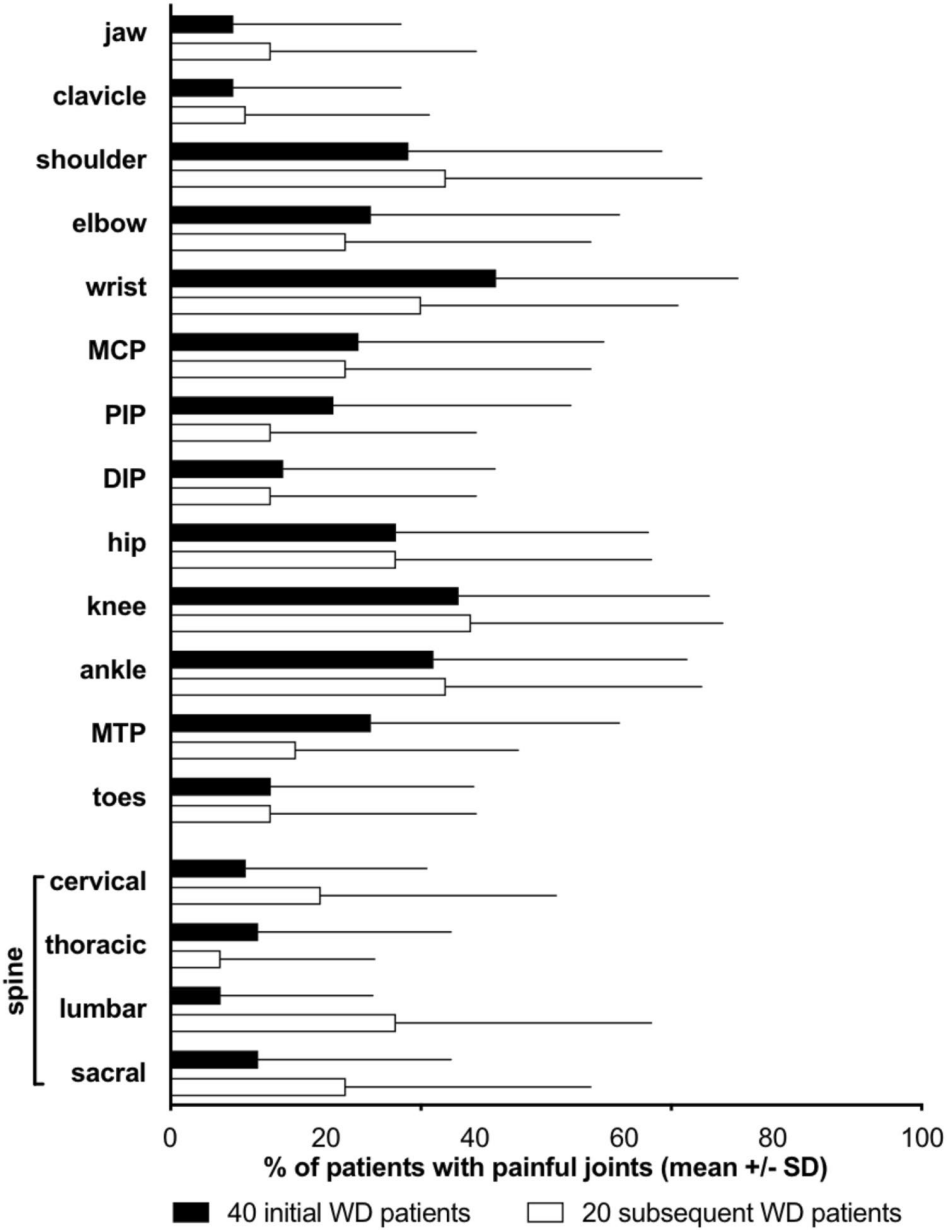
Percentage of Whipple’s disease patients with painful joints in the initial and subsequent series. *PIP* proximal interphalangeal joints, *DIP* distal interphalangeal joints, *MCP* metacarpo-phalangeal joints, *MTP* metatarsophalangeal joints.

All patients diagnosed with Whipple’s disease met the diagnostic criteria^12,13^. The initial group of 40 patients with Whipple’s disease consisted of 28 male and 12 female patients; mean age of 57.6 ± 11.0 years (M ± SD), the subsequent series of 20 patients, devised as validation group, consisted of 13 male and seven female patients, aged 61.5 ± 12.04 years (M ± SD) not significantly different from the initial group (p = 0.13). Clinical characteristics were also similar in the two groups (Table 1, Fig. 1).

**Table 1:** Baseline clinical characteristics in the two groups of patients with Whipple’s disease.

Characteristic	Initial 40		Subsequent 20		
	Tested, n	n (%)	Tested, n		n (%)
Diarrhea/malabsorption	40	19 (48)	20		9 (45)
Weight loss	40	19 (48)	20		12 (60)
Arthritis	40	34 (85)	20		18 (90)
Central nervous symptoms	40	5 (13)	20		5 (25)
Fever	40	9 (23)	20		5 (30)
Initial *T. whipplei*-specific PCR, positive in the cerebrospinal fluid	32	17 (50)	18		6 (30)
PAS-positive staining in duodenal mucosa	40	25 (63)	20		14 (70)
PAS-positive cells of type I (von Herbay), in duodenal mucosa	40	21 (53)	20		12 (60)
*T. whipplei*-specific IHC^a^, positive in duodenal mucosa	39	32 (82)	20		19 (95)
*T. whipplei*-specific PCR, positive in duodenal mucosa	29	25 (86)	7		6 (86)
*T. whipplei-specific* PCR, IHC, or PAS positive in synovial tissue/fluid^b^	7	7 (100)	5		5 (100)
*T. whipplei*-specific PCR, positive in a cardiac valve	1	1 (100)	1		1 (100)

Significance (Fisher’s exact test): not signisicant for all parameters.

^a^IHC: specific immuno-histochemistry.

^b^Detection of *T. whipplei* in synovial fluid by PCR and in synovial tissue by PAS staining and IHC.

The 30 patients diagnosed with rheumatoid arthritis consisted of eight males and 22 females; mean age of 58.2 ± 14.5 years (M ± SD); 25 patients with axial spondyloarthritis (18 male, seven female), mean age of 50 ± 14.8 years (M ± SD); 21 with psoriatic arthritis (12 male, nine female), mean age 48.8 ± 15.65 years (M ± SD); and 15 patients with symptoms of palindromic arthritis (six male, eight female), mean age 59.9 ± 9.19 (M ± SD). The inclusion rate of patients diagnosed with palindromic rheumatism was higher than 90% and was higher than 80% in patients with rheumatoid arthritis, psoriatic arthritis, and axial spondyloarthritis. Recruitment rates lower than 100% were not due to selected patient characteristics but due to organizational problems, such as absence of the investigator when the patient was in the outpatient department or language barrier.

Patients with Whipple’s disease were treated with intravenous ceftriaxone followed by oral trimethoprim/sulfamethoxazole (TMP/SMX) for 12 months (n = 33), n = 1 for three months), with oral doxycycline plus hydroxychloroquine (n = 23), with ceftriaxone followed by 12 months oral doxycycline plus hydroxychloroquine (n = 2), with oral doxycycline followed by TMP/SMX or intravenous penicillin each in one patient. Patients with rheumatic conditions in Baden-Baden received standard treatment for their specific conditions.

The time from first appearance of symptoms up to time of diagnosis was part of the questionnaire in patients with Whipple’s disease. In 24 of the 40 patients an answer was obtained. Mean diagnostic delay in this group was 7 ± 4.5 years M ± SD with a range of 0–15 years. This issue of diagnostic delay was part of the questionnaire for patients with Whipple’s disease only. Otherwise all questionnaires were identical.

Painful articular manifestation in patients with Whipple’s disease was bilateral and symmetrical in 22 cases. In 14 patients, some joints were affected unilaterally, others bilaterally, two patients had only unilateral symptoms and two patients had no arthritis but symmetrical musculotendinous symptoms.

Anatomical localization of pain in the joints and pain at the spinal regions of the initial 40 and the subsequent series of 20 patients with Whipple’s disease are displayed in Fig. 1.

Total number of painful joints, shown in Table 2, was similar in patients with psoriatic arthritis, palindromic arthritis, rheumatoid arthritis, axial spondyloarthritis, and Whipple’s disease (tested not significant by one-way ANOVA, Table 2). But a more detailed study, shown in Table 3, indicates that the incidence of pain localized in the spinal and sacroiliac regions was significantly more common in patients with axial spondyloarthitis compared to patients with psoriatic arthritis, palindromic arthritis, rheumatoid arthritis, and Whipple’s disease (tested significant by ordinary one-way ANOVA that revealed a p-value of < 0.0001; Table 3).

**Table 2:** Number of painful joints in patients with psoriatic-, palindromic-, rheumatoid-, and axial spondyloarthritis compared to the initial 40 patients with Whipple’s disease.

	Psoriatic arthritis	Palindromic arthritis	Rheumatoid arthritis	Whipple’s disease	Axial spondylo-arthritis
Patients (n)	21	15	30	40	25
Joints involved (n)	95	88	199	208	113
Mean	4.524	5.867	6.633	5.200	4.520
Standard deviation	± 2.695	± 2.356	± 3.306	± 3.368	± 2.568
p-value*	0.758*	0.758*	0.188*		0.758*

*One-way ANOVA followed by Holm-Sidak´s multiple comparison tests vs. Whipple’s disease patients.

**Table 3:** Frequency of spinal localization and episodic character of pain in 40 patients with Whipple’s disease compared to palindromic-, rheumatoid -, psoriatic -, and axial spondyloarthritis.

	p-value one-way ANOVA	Psoriatic arthritis	Palindromic arthritis	Rheumatoid arthritis	Whipple’s disease	Axial spondylo-arthritis
Spinal localization of pain	< 0.0001*	14.29%	6.67%	6.67%	10.00%	56.00%
SD		± 35.86	± 25.82	± 25.37	± 30.38	± 50.66
p-value		< 0.0001**	< 0.0001**	< 0.0001**	< 0.0001**	
Episodic pain, once a week	0.0005*	14.29%	46.67%	20.00%	52.50%	12.00%
SD		± 35.86	± 51.64	± 40.68	± 50.57	± 33.17
p-value		0.0042***	ns***	0.0047***		0.0015***
Insidious beginning	ns*	61.90%	40.00%	60.00%	65.00%	68.00%
SD		± 49.76	± 50.71	± 49.83	± 48.30	± 47.61
Rapid beginning	ns*	28.57%	60.00%	43.33%	25.64%	28.00%
SD		± 46.29	± 50.71	± 50.40	± 44.24	± 45.83

*SD* standard deviation.

*One-way ANOVA.

**One-way ANOVA followed by Holm–Sidak’s multiple comparison tests vs. axial spondyloarthritis patients.

***One-way ANOVA followed by Holm–Sidak’s multiple comparison tests vs. Whipple’s disease patients.

In total, 25 clinical variables were recorded in each patient (see “Supplemental questionnaire”). Episodic pain occurring about once a week with rapid onset, springing from joint to joint (saltatory pain) was most common and conspicuous in patients with palindromic arthritis, second most common in patients with Whipple’s disease. To distinguish Whipple’s disease and palindromic arthritis, pain with rapid onset occurring in nine out of 15 patients with palindromic arthritis and in ten out of 40 patients with Whipple’s disease and pain affecting the same joints occurring in 0 out of 15 patients with palindromic arthritis and in nine out of 40 patients with Whipple’s disease seemed to be most suitable in the differential diagnosis (Table 3). Onset of the painful attacks in patients with Whipple's disease was somewhat delayed (insidious onset, versus rapid onset) and the saltatory character not as distinct as in patients with palindromic arthritis. 

Episodic pain in the patients with rheumatoid arthritis, psoriatic arthritis, and axial spondyloarthritis was uncommon; pain in these disorders was fluctuating at the typical locations (Table 3). Joint pain in patients with Whipple’s disease predominantely occurred in the large central joints including the wrists and rarely in the small peripheral joints, such as the toes (Fig. 1, Table 4).

**Table 4:** Multiple logistic regression analysis of symptoms in 40 patients with Whipple’s disease versus patients with palindromic arthritis, rheumatoid arthritis, psoriatic arthritis, and axial spondyloarthritis.

	Odds ratio [odds ratio after backward selection]	95% CI [95% CI after backward selection]
Whipple’s disease versus the four rheumatic disorders tested		
Male	3.408 [3.910]	1.299–8.942 [1.552–9.854]
Pain once per week	3.269 [3.434]	1.149–9.297 [1.284–9.183]
Episodic pain	2.584 [3.488]	0.836–7.990 [1.212–10.039]
Pain affecting same joints	0.690	0.091–5.216
Toes	0.390	0.136–1.116
Saltatory pain	1.051	0.143–7.705
Ankle pain	1.805	0.693–4.703
Whipple’s disease versus palindromic arthritis		
Pain with rapid onset	0.175 [0.167]	0.043–0.708 [0.042–0.654]
Pain affecting same joints	559,853,342.0^a^ [1155605404.0^a^**]**	0.000 [0.000]
Shoulder	0.357	0.075–1.700
Whipple’s disease versus rheumatoid arthritis		
Male	11.982 [14.285]	1.738–82.620 [2.166–94.215]
Pain once per week	1.216	0.150–9.852
Episodic pain	9.806 [9.184]	1.203–79.951 [1.625–51.919]
Rapid onset of pain	0.682	0.133–3.499
Pain affecting same joints	0.750	0.048–11.714
Toes	0.052 [0.058]	0.007–0.396 [0.008–0.397]
Whipple’s disease versus psoriatic arthritis		
Pain once per week	2.431	0.452–13.064
Episodic pain	4.831 [7.298]	1.045–22.342 [1.938–27.487]
Pain affecting same joints	0.953	0.047–19.142
Toes	0.149 [0.138]	0.024–0.923 [0.024 – 0.805]
Saltatory pain	1.081	0.052–19.919
Shoulder	8.170 [10.639]	1.002–66.643 [1.517–74.597]
Whipple’s disease versus axial spondyloarthitis		
Pain once per week	1.554	0.194–12.493
Episodic pain	10.689 [15.035]	1.056–108.151 [1.711–132.100]
Affecting same joints	0.862	0.101–7.346
Saltatory pain	2.928	0.348–24.633
Sacral and ileosacral pain	0.013 [0.016]	0.001–0.158 [0.001–0.171]
Ankle pain	24.122 [26.757]	1.910–304.680 [2.436–293.874]

Values greater than 1.0 suggest a constellation of symptoms favoring Whipple’s disease, lower values are indicative for one of the other disorders.

^a^There was no patient in the palindromatic group in whom the same joints were affected in the following attacks.

**Figure 2:**
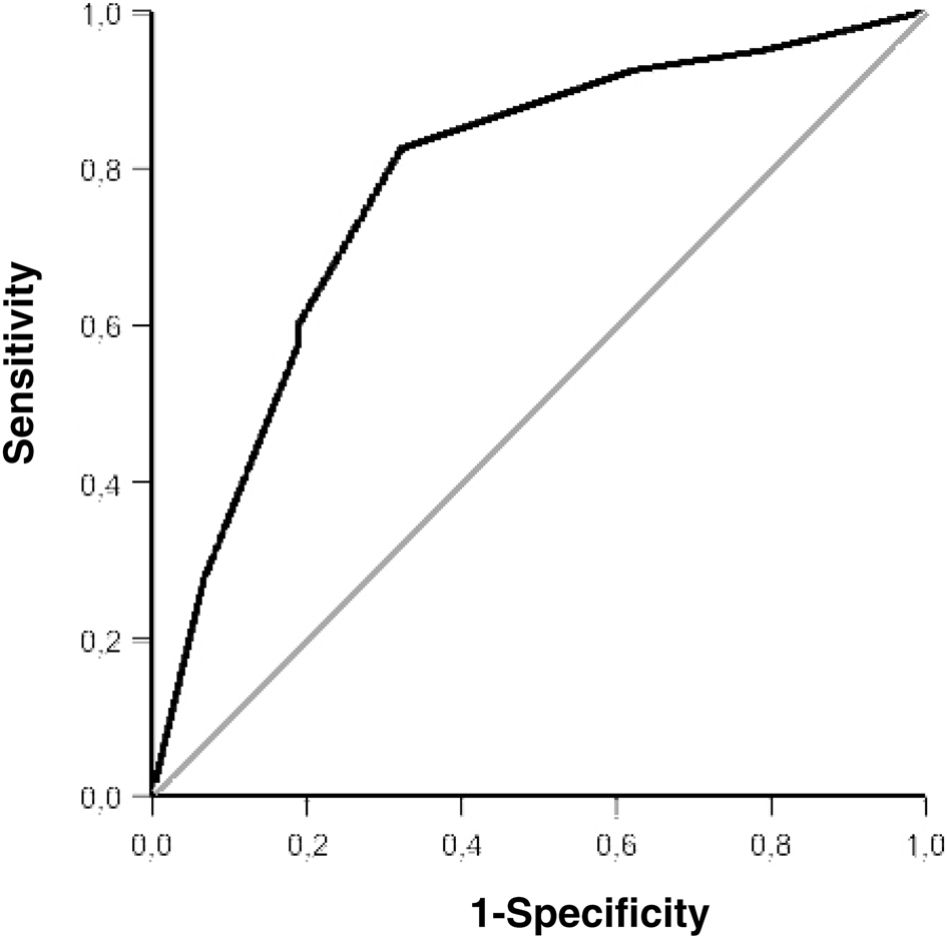
Discriminatory ability of three clinical characteristics for the diagnosis of Whipple’s disease: male, episodic pain occurring about once per week in patients with Whipple’s disease plotted against patients with palindromic arthritis, rheumatoid arthritis, psoriatic arthritis, and axial spondyloarthritis in a ROC curve of the predicted value from a multiple logistic regression model p < 0.001. Data for the other disorders see Table 4.

Inserting the regression parameters of Table 4 in the multiple logistic equation resulted in a logistic equation predicted probability for the diagnosis of Whipple's disease of 43.4% (SD ± 0.19%) versus a significantly lower logistic equation predicted probability of 23.8% (SD ± 0.19%) for this diagnosis in the aggregate of patients with palindromic arthritis, rheumatoid arthritis, psoriatic arthritis, and axial spondyloarthritis. The mean area under the curve ± standard deviation was 0.781 ± 0.044, 95% confidence interval was 0.695–0.867, the asymptotic significance p < 0.001. The area under the curve of 0.781 resulting from the logistic regression model indicates moderate accuracy (Fig. 2).

Using the regression parameters obtained in the subsequent series of 20 patients with Whipple’s disease, devised as validation group, resulted in a mean probability for the diagnosis of Whipple’s disease of 38.2 ± 0.28% (M ± SD), which was not significantly different from the result obtained in the initial series of 40 patients of 43.4 ± 0.19% (p = 0.376).

**Figure 3:**
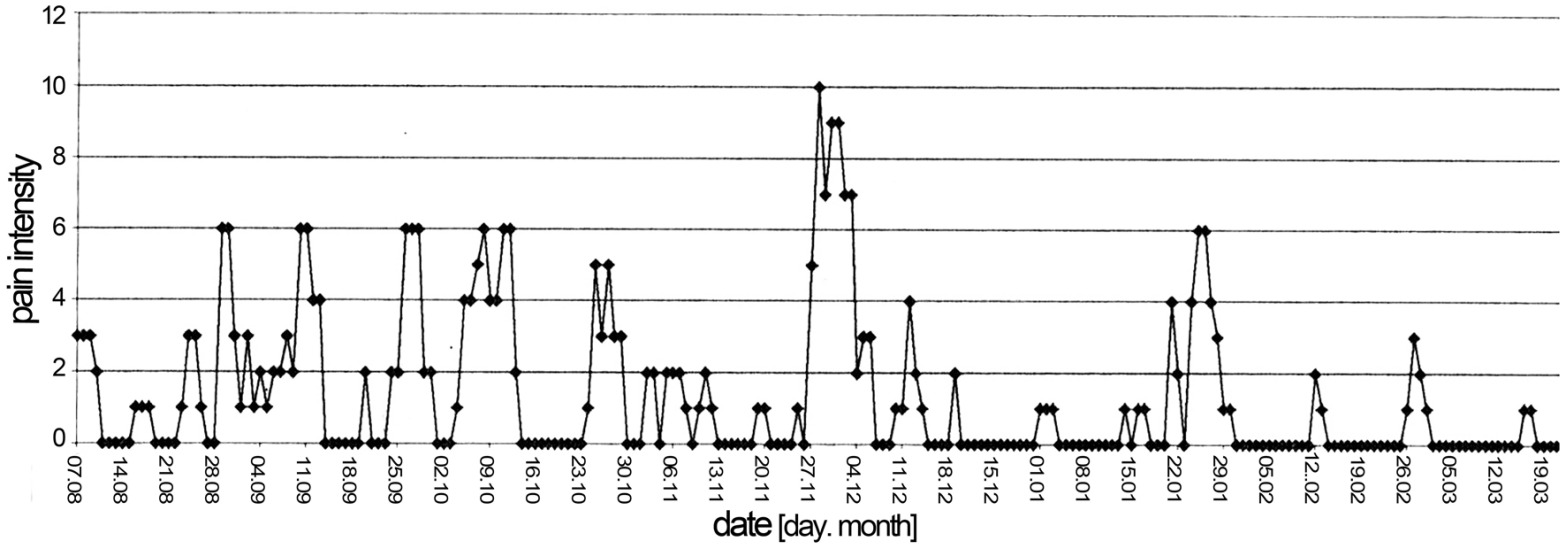
Musculotendinous pain, recorded by a patient with Whipple’s disease during a period of 7 months. Pain intensity on the y axis ranged from zero, indicating no pain to ten indicating maximal pain. Details see text.

Figure 3 depicts musculotendinous pain recorded and plotted by a 74-year old patient with Whipple’s disease, patient no. 14 of the present series. The patient described saltatory painful attacks erratically hitting the neck, the back of the right and left hand, the lower back and lumbar regions, right and left upper arms, both scapular regions, middle thorax, right and left wrists, and the instep of the left and right foot, lasting usually for 1 up to 3 days. Seronegative rheumatoid arthritis was diagnosed and treatment with oral prednisone at a dose of 20 mg given during August, followed by 10 mg until September; thereafter prednisone was stopped and weekly parenteral 10 mg methotrexate was given up to April. The rheumatic attacks abated in March when suddenly, from one day to the other, severe diarrhea began continuing up to the time the diagnosis of Whipple’s disease was made and appropriate antibiotic treatment was initiated.

## Discussion

This is a prospective study investigating the differential diagnostic value of rheumatic symptoms in patients with Whipple’s disease compared to patients with rheumatoid arthritis, axial spondyloarthritis, psoriatic arthritis, and palindromic arthritis, the latter important as previous observations have suggested the clinical manifestations in Whipple’s disease to be similar in the patients diagnosed with palindromic rheumatism^2,3,7,19^.

One should be aware that the data on which our conclusions are based are not precise laboratory measurements but answers of laypersons, i.e. patients, supplied by written questionnaires. Calculation of standard deviations and logistic regression analysis under these circumstances may represent a form of pseudo-exactness.

It may be more reasonable to consider the results as indicating trends, useful to generate a new hypothesis to be tested in further projects.

Several findings in the present study, however, indicate plausibility: patient characteristics show that rheumatoid arthritis was more frequent in females as reported in the literature^20^, whereas the reverse, also as expected, was true in patients with axial spondyloarthritis and in Whipple’s disease^15,17,18,21^. Further, the mean age of 57 years in the patients with Whipple’s disease in the present study was in agreement with the age in the cohorts with this disorder of previous studies^13,21–23^.

Although the total number of involved joints, as shown in Table 2, was not discriminatory, plausibility is supported by the findings of Table 3, showing that pain in the spinal regions was recorded significantly more often by patients with axial spondyloarthritis than by patients with any of the other rheumatic disorders including Whipple’s disease and by Table 3, indicating that the peculiar episodic character of joint pain in patients with palindromic arthritis and in patients with Whipple’s disease was reproduced. Onset of painful attacks was more rapid in patients with palindromic arthritis.

Logistic regression analysis disclosed that a combination of clinical characteristics was able to discriminate patients with Whipple's disease from patients with rheumatoid arthritis, axial spondyloarthritis, psoriatic arthritis, and palindromic arthritis. A constellation of episodic attacks lasting for about a week, involving sometimes the same joints and sparing the toes and to some degree distal finger joints in males was able to significantly differentiate patients with Whipple's disease from the four rhematic disorders (Table 4, Fig. 2). In this table, values greater than 1.0 suggest a symptom constellation favoring Whipple's disease, lower values are indicative for one of the other disorders.The most difficult clinical differential diagnosis was Whipple’s disease versus palindromic arthritis. Painful episodes beginning insidiously and affecting repeatedly the same joints were more characteristic in Whipple’s disease, whereas pain episodes beginning rapidly and springing briskly from one joint to the other were more characteristic in palindromic rheumatism (Tables 3 and 4).

Episodic attacks of pain always favored the diagnosis of Whipple’s disease, whereas painful affection of the toes and pain in the sacroiliac region would have been unusual (Table 4). The spectrum of clinical characteristics determined in the intial 40 patients with Whipple's disease did not differ significantly from the characteristics in the subsequent and independent series of 20 patients of the validation group. Anatomical localization of joint pain in both groups is shown in Fig. 1.

Several limitations, however, must be considered:

While the patients with Whipple’s disease were successively recruited and therefore unselected, the patients in the control groups with classic rheumatic disorders were selected to match the respective diagnostic criteria.

It is known, however, that as many as 25% of patients with rheumatic symptoms, in particular seronegative cases, cannot be diagnosed definitively and may remain undiagnosed during 5–10 years of follow-up^3,24^.

Such a group of patients with undifferentiated arthritis, including some with the ill-defined, so-called overlap syndromes, as control subjects was not available for the actual study. A diagnosis of Whipple’s disease in such a diverse group would have been more demanding.

Larger numbers of patients in each group would have been helpful in establishing accurate mean data. Such averaged values, however, would be of little use for the differential diagnosis in individual patients when signs and symptoms in a specific case may deviate widely from the mean.

Patient no. 14 with Whipple’s disease, who recorded clinical exacerbations on a pain scale for several months, noted that overlapping and cumulating short painful episodes in various locations led to the impression of painful courses lasting sometimes longer than 1 week (Fig. 3). It is not clear whether the abatement of musculotendinous pain in March in this patient was due to previous treatment with methotrexate and prednisone or whether it occurred spontaneously. Sudden onset of diarrhea at the time joint pain is abating after termination of immunosuppressive therapy in patients with Whipple’s disease has, however, been described previously^5^.

This patient was one of two in the present series of 60 patients with Whipple’s disease who did not have arthritic but musculotendinous pain. The answers given in the questionnaires (Table 3) and the literature^2,3,7^ indicate that pain spikes lasting for a few days are characteristic of Whipple’s disease, irrespective of whether arthritic or musculotendinous pain attacks prevail. Similar but even sharper articular pain spikes can be registered in patients with palindromic rheumatism.

A clinical entity of palindromic rheumatism or palindromic arthritis is not established. Nevertheless, patients with palindromic types of joint pain do occur, frequently delaying or defying a specific diagnosis. In this particular group of patients, heightened awareness is necessary to exclude Whipple’s disease arthritis.

The episodic character of arthritis in Whipple’s disease, described previously^2,3^ was recorded in our series in 52% of the patients (Table 3). Whether this magnitude is representing reality or is due to the imponderables of the questionnaire technique remains an open question. The fact that episodic arthritis, the definition for patients with palindromic arthritis, was recorded also in only 46% of the patients with this disorder indicates that the questionnaire technique rather poorly depicts quantitative aspects. However, all patient groups were evaluated in the same way and episodic pain was recorded in the patients with psoriatic arthritis, rheumatoid arthritis, and axial spondyloarthritis in rates of only 14%, 20% and 12% of the cases, respectively, i.e. in significantly lower rates (Table 3).

Episodic arthritis as a clinical phenomenon also occurs in crystal-induced forms of arthritis, such as CPPD crystal-induced arthritis (chondrocalcinosis) or gout^25^. Such a differential diagnosis must be carefully considered in patients with this type of clinical manifestation. In these patients, synovial tissue or fluid obtained from painful joints should not only be examined for the characteristic crystals but, if negative, with a specific PCR for *T. whipplei.*

The abrupt onset of inflammatory symptoms in patients with Whipple’s disease at the beginning of a painful episode is reminiscent of the abrupt onset of inflammatory symptoms in patients with Whipple’s disease developing immune reconstitution inflammatory syndrome (IRIS) within 24 h upon institution of an effective antibiotic treatment^4,26^. This crescendo-type of inflammation has been discussed to be related to rapid reactivation of the T-cell system leading to a “storm” of pro-inflammatory cytokine release^4,26^. It remains to be determined whether the stormy and episodic courses of inflammation in untreated patients with Whipple’s disease are also related to fluctuating T-cell activity.

In conclusion, our findings suggest that all patients with anti-CCP and/or rheumatoid factor-negative variants of palindromic types of arthritis, when crystal-induced diseases cannot be established, should be carefully examined for Whipple’s disease regardless of presence or absence of diarrhea.

Our study, employing logistic regression analysis for the differential diagnosis of Whipple’s disease versus some common rheumatic disorders, providing odds ratios (Table 4) and a ROC curve (Fig. 2), if approved, may be used as a predictive algorithm for the diagnosis of this rare disorder, complementary to clinical decision-making^27^.

It is evident from our study, however, that not all patients with Whipple’s disease run a clinically apparent “palindromic” course and that about one third of the patients are female^11^. In these patients a diagnosis will remain challenging.

It was not the aim of the present investigation to study the value of general laboratory tests in the diagnosis of Whipple’s disease. Acute-phase responses are activated in rheumatic diseases as well as Whipple’s disease, whereas determination of rheumatoid factors can be diagnostic. Our experience and those of others^6,9^ indicate that periodic acid-Schiff (PAS) stain or polymerase chain reaction (PCR) for *T. whipplei* in small bowel mucosal biopsies may not be sufficient to either diagnose or exclude Whipple’s disease in a particular patient. In only about 50% of the patients with Whipple’s disease in the present study, the pathognomonic PAS positive macrophages subtype 1 were detected in the duodenal mucosal biopsy (Table 1). More promising is a *T. whipplei* -specific PCR performed in synovial fluid or tissue of affected joints^6^. Symptoms do have their value in the decision as to which patient should be examined and at which joint.

Logistic regression analysis disclosed that a combination of clinical characteristics was able to discriminate patients with Whipple’s disease from patients with rheumatoid arthritis, axial spondyloarthritis, psoriatic arthritis, and palindromic arthritis.

We found no single clinical marker unequivocally indicating the diagnosis in Whipple’s disease but a spectrum of symptoms warranting further diagnostic testing. The constellation of a male patient with episodic and saltatory bouts of painful arthritis of about one week duration but sometimes confluent for a longer time, with pain beginning insidiously, often repeatedly in the same joints, sparing the toes and—to some degree—also the distal finger joints was the typical clinical appearance of a patient with Whipple’s disease.

## Supplementary Information

Supplementary Information.

## Data Availability

A sample of the questionnaires (in German) is available from the corresponding author on request. The signed consent form of each patient is in the patient’s hospital or praxis chart and can be retrieved from there. The original of the consent to use the pain scale recording for publication is available from the first author on request. All data generated or analysed during this study are included in this published article.
