# Supplementary material for: Differential diagnostic value of rheumatic symptoms in patients with Whipple’s disease
Source: Sci Rep. 2021 Mar 16;11:5980. doi: 10.1038/s41598-021-85217-2 (PMC7966399; doi:10.1038/s41598-021-85217-2)
Supplement: Supplementary file 1 — Supplementary Information. [file 41598_2021_85217_MOESM1_ESM.docx]

**Differential diagnostic value of rheumatic symptoms in patients with Whipple’s disease.**

Gerhard E. Feurle MD., Verena Moos Ph. D., Andrea Stroux dipl. Math., Nadine Gehrmann-Sommer, Denis Poddubnyy MD., Christoph Fiehn MD., Thomas Schneider MD. Ph. D.

Supplementary Information:

**Whipple’s disease – Rheumatism Questionnaire**

In this questionnaire we ask you to describe and write down the kind of pain in the joints

and if present in the adjacent muscles and tendons.

1. What was the overall course of joint pain?

I did have no joint pain O

The pain did mostly affect the same joints O

The pain did usually or always affect different joints, “jumping” from one joint

to another O


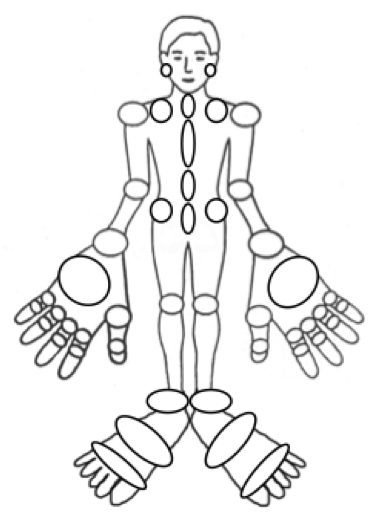
2) Please mark in the figure the usual and main locations of joint pain:

Please mark particular describe if required:

affected joints

with a bold cross: _________________________

_________________________

_________________________

_________________________

_________________________

_________________________

_________________________

_________________________

_________________________

_________________________

_________________________

3) Did the joint pain occur or arise from time to time in events?

Yes O

No, the pain was rather continuous and long lasting O

4) If the pain occurred from time to time, what was the time course?

a) about once per week O

b) about once per month O

c) more rarely O

d) if you are not sure, please explain and describe:

________________________________________________________________________________________________________________________________________________________________________________________________________________________________________________________________________________________________

5) How was the onset of joint pain?

rapid beginning O

slow beginning O

6) If the pain occurred from time to time, how long did the pain attacks last?

a) hours O

b) days O

c) weeks O

d) months O

e) if you are not sure, please explain and describe:

________________________________________________________________________________________________________________________________________________________________________________________________________________________________________________________________________________________________

7) Did you have pain in the soft tissues, the muscles and tendons away from the joints?

Yes O

No O

Please describe:

________________________________________________________________________________________________________________________________________________________________________________________________________________________________________________________________________________________________

Thank you very much for your help! If you want to comment, this would be welcome:

________________________________________________________________________________________________________________________________________________________________________________________________________________________________________________________________________________________________

________________________________________________________________________________________________________________________________________________________________________________________________________________________
